# Dynamic perioperative CA19-9 and inflammatory-nutritional recovery patterns are associated with early recurrence and survival after resection for pancreatic ductal adenocarcinoma: a real-world cohort study

**DOI:** 10.3389/fmed.2026.1899908

**Published:** 2026-09-16

**Authors:** Chengyu Wang, Guoping Zhong, Anju Cao, Juan Li, Hang Jiang

**Affiliations:** Department of General Surgery, The Third People’s Hospital of Yunnan Province, Kunming, China

**Keywords:** CA19-9, early recurrence, pancreatic ductal adenocarcinoma, prognostic nutritional index, real-world cohort, recurrence-free survival, systemic inflammation

## Abstract

**Background:**

Early recurrence after resection for pancreatic ductal adenocarcinoma (PDAC) remains common and is not fully captured by conventional clinicopathologic variables. This study evaluated whether dynamic perioperative CA19-9 and inflammatory-nutritional recovery patterns were associated with early recurrence and survival after curative-intent resection.

**Methods:**

This real-world cohort study included patients with pathology-confirmed PDAC who underwent curative-intent R0/R1 resection at The Third People's Hospital of Yunnan Province. Postoperative biomarker assessments were defined at postoperative days (PODs) 21–45 and 75–105. A transparent three-level dynamic pattern integrated early CA19-9 behavior with 3-month NLR, CRP, and PNI recovery; 255 of 268 patients were classifiable. Because exposure ascertainment extended through POD 105, exposure-dependent analyses used a common POD-105 landmark. The primary outcome was recurrence within 12 months; secondary outcomes were recurrence-free survival (RFS) and overall survival (OS). Multivariable regression, 1,000-replicate bootstrap internal validation, and competing-risk sensitivity analysis were used.

**Results:**

Among 268 included patients, 78 (29.1%) experienced early recurrence, 149 (55.6%) developed recurrence during follow-up, and 130 (48.5%) died. In the primary dynamic-pattern cohort (*n* = 255), 94 patients (36.9%) were classified as favorable, 121 (47.5%) as intermediate, and 40 (15.7%) as poor; early recurrence occurred in 20.2%, 28.1%, and 50.0%, respectively. Compared with the favorable pattern, the adjusted odds ratios for early recurrence were 2.02 (95% CI, 1.01–4.02) for the intermediate pattern and 4.96 (95% CI, 2.10–11.71) for the poor pattern. The poor pattern was also associated with shorter RFS (adjusted HR, 2.12; 95% CI, 1.31–3.42) and OS (adjusted HR, 2.52; 95% CI, 1.50–4.24). The apparent AUC was 0.648 for the clinical model and 0.702 after adding the dynamic pattern (DeLong *P* = 0.057); corresponding optimism-corrected AUCs were 0.605 and 0.661.

**Conclusion:**

A poor postoperative dynamic recovery pattern was consistently associated with higher early-recurrence risk and shorter RFS and OS after PDAC resection. The dynamic pattern provided modest incremental discriminative information beyond clinicopathologic variables, but performance remained moderate after internal validation. External validation is required before clinical implementation.

## 1 Introduction

Pancreatic ductal adenocarcinoma (PDAC) remains one of the most lethal solid tumors worldwide. Global cancer statistics continue to show a high mortality-to-incidence ratio for pancreatic cancer, reflecting late diagnosis, aggressive biology, and limited durability of local treatment (1, 2). Although surgical resection offers the only established route to long-term survival, only a minority of patients present with resectable disease, and recurrence after pancreatectomy is frequent even when resection is macroscopically complete (3–5).

Modern systemic therapy has improved outcomes in selected patients. Adjuvant gemcitabine prolonged disease-free survival in the CONKO-001 trial, gemcitabine plus capecitabine improved survival in ESPAC-4, and modified FOLFIRINOX produced superior outcomes in fit patients after resection (6–8). Neoadjuvant strategies are also increasingly used for borderline resectable disease and selected resectable tumors, although the balance between early systemic control and timely surgery remains individualized (9, 10). Even with these advances, recurrence within the first postoperative year remains a clinically important event because it often reflects occult systemic disease and limited benefit from surgery alone (11, 12).

Several clinicopathologic factors, including nodal involvement, margin status, lymphovascular invasion, and tumor grade, have been linked to recurrence and long-term survival after PDAC resection (13, 14). These factors are informative, but most are static descriptors of tumor burden at the time of surgery. They do not fully capture how residual disease and host condition evolve during the early postoperative period, when decisions about adjuvant treatment and surveillance intensity are usually made.

CA19-9 is the most widely used serum biomarker in PDAC. Earlier studies showed that perioperative CA19-9 levels and post-resection CA19-9 values were associated with recurrence and survival (15, 16). Later work refined this concept by showing that postoperative surveillance CA19-9 and normalization after treatment may change prognosis over time rather than simply mirror baseline tumor burden (17, 18). More recently, CA19-9 response dynamics and normalization during multimodal therapy have been associated with postoperative outcomes, supporting a longitudinal rather than single-time-point interpretation of this marker (19). However, CA19-9 is not tumor-specific, can be influenced by biliary obstruction, and may be uninformative in suspected non-secretors, so it is rarely sufficient as a standalone risk tool (20, 21).

Host recovery may provide a complementary signal. Systemic inflammation and malnutrition are closely linked to cancer outcomes, treatment tolerance, and postoperative recovery (22). In PDAC, the neutrophil-to-lymphocyte ratio (NLR), prognostic nutritional index (PNI), and systemic immune-inflammation index (SII) have each been studied as prognostic markers, and meta-analyses support their association with survival in pancreatic cancer or resected pancreatic cancer populations (23–27). Yet most available studies evaluate preoperative or single postoperative measurements, leaving the clinical value of recovery trajectories less well defined.

We therefore designed a real-world cohort study to evaluate whether dynamic perioperative CA19-9 patterns combined with inflammatory-nutritional recovery were associated with 12-month early recurrence and subsequent survival after curative-intent resection for PDAC, and to quantify their incremental risk-stratification information beyond conventional clinicopathologic variables.

## 2 Methods

### 2.1 Study design and population

This was a single-center real-world cohort study conducted at the Department of General Surgery, The Third People's Hospital of Yunnan Province, Kunming, China. The study followed the principles of the STROBE statement for observational studies and used TRIPOD-aligned reporting elements for the prognostic modelling components (28, 29).

Patients undergoing surgery between March 5, 2016 and December 31, 2024 were screened from a hospital surgical cohort of pathology-confirmed pancreatic malignancy assessed for surgical treatment. Eligible patients had pathology-confirmed PDAC and underwent curative-intent R0/R1 resection. Patients were excluded if they had non-PDAC pathology or periampullary non-PDAC tumors, palliative bypass or exploration without curative-intent resection, perioperative death before planned biomarker follow-up, suspected CA19-9 non-secretor/low-expressor status, incomplete serial-marker data, or follow-up shorter than 12 months without recurrence. Of the 28 patients excluded for CA19-9/serial-marker eligibility, 15 were suspected non-secretors/low expressors and 13 had incomplete serial-marker data. The full eligible cohort included 268 patients. Thirteen additional patients lacked 3-month CRP, which was required for the three-domain recovery definition, and therefore could not be assigned a dynamic pattern; exposure-dependent primary analyses included 255 patients.

### 2.2 Ethics and data source

The dataset was obtained from The Third People's Hospital of Yunnan Province, where all included patients were treated. The study was approved by the Ethics Committee of The Third People's Hospital of Yunnan Province (approval number ynth1251103). Because the analysis used de-identified retrospective clinical data, the requirement for individual informed consent was waived by the approving committee.

### 2.3 Clinical variables and biomarkers

Baseline variables included age, sex, body mass index, smoking and alcohol history, diabetes, new-onset diabetes, hypertension, American Society of Anesthesiologists class, tumor location, resectability status, neoadjuvant therapy, jaundice, preoperative biliary drainage, operative procedure, vascular resection, tumor size, pathologic T and N stage, TNM stage group, histologic grade, lymphovascular invasion, perineural invasion, margin status, lymph nodes examined, positive lymph nodes, lymph node ratio, operation time, estimated blood loss, clinically relevant postoperative pancreatic fistula, postoperative infection, Clavien-Dindo grade III or higher complications, postoperative length of stay, and adjuvant chemotherapy.

Laboratory markers included preoperative bilirubin, albumin, C-reactive protein (CRP), neutrophil count, lymphocyte count, platelet count, NLR, platelet-to-lymphocyte ratio, SII, PNI, CA19-9, carcinoembryonic antigen, and CA125, together with postoperative albumin, CRP, NLR, SII, PNI, and CA19-9 values. For analysis, the 1-month assessment window was POD 21-45 with POD 30 as the target, and the 3-month window was POD 75-105 with POD 90 as the target. When more than one measurement was available within a window, the specimen closest to the target day was selected; a same-day panel was preferred, and CBC/CRP/albumin obtained within ±3 days of CA19-9 was treated as the same visit. CA19-9 normalization at 1 month was defined as CA19-9 ≤37 U/mL; a marked decline was defined as a ≥50% decrease from the preoperative value to 1 month. A 3-month rebound was defined as a 3-month/1-month CA19-9 ratio ≥1.50 together with a 3-month CA19-9 concentration >37 U/mL. Suspected CA19-9 non-secretor/low-expressor status was defined phenotypically from serial measurements: 10 patients had preoperative, 1-month, and 3-month CA19-9 values consistently ≤7 U/mL, and 5 had persistently low values between 7 and 15 U/mL that were considered uninformative for longitudinal kinetics. Lewis antigen and FUT3 testing were not available, so these cases were not considered genetically confirmed non-secretors. Because CA19-9 may be unreliable in non-secretors and can be affected by cholestasis, patients with suspected non-secretor/low-expressor status or incomplete serial CA19-9 data were not included in the dynamic biomarker cohort (20, 21).

Inflammatory-nutritional recovery was defined using three 3-month domains: NLR ≤5, CRP ≤10 mg/L, and PNI ≥45. Recovery was classified as good when all three criteria were met, intermediate when exactly two were met, and poor when zero or one was met. Here, recovery denotes attainment of favorable 3-month state thresholds rather than a formally modeled longitudinal trajectory. For CA19-9, normalization was defined as a 1-month value ≤37 U/mL, a marked decline as a ≥50% decrease from the preoperative value to 1 month, and rebound as a 3-month/1-month ratio ≥1.50 together with a 3-month value >37 U/mL. The CA19-9 state was classified as favorable when normalization or a marked decline was present without rebound, and adverse otherwise. The composite dynamic pattern was classified as favorable when a favorable CA19-9 state co-occurred with good or intermediate recovery, poor when an adverse CA19-9 state co-occurred with poor recovery, and intermediate for all remaining classifiable discordant combinations. Because classification required complete 3-month NLR, CRP, and PNI, 13 patients with unavailable 3-month CRP were not assigned a dynamic pattern, yielding 255 classifiable patients. Operational definitions are summarized in Supplementary Table S1.

### 2.4 Outcomes

The primary outcome was early recurrence, defined as documented recurrence after the POD-105 landmark and within 365 days after surgery. Recurrence was established from clinical records and cross-sectional imaging rather than CA19-9 alone. RFS was defined from the POD-105 landmark to the first recurrence or death from any cause, whichever occurred first; patients without an event were censored at last follow-up. OS was defined from the POD-105 landmark to death from any cause. Follow-up was administratively censored on May 31, 2026. Postoperative surveillance included a surgical review at approximately POD 10–21, a 1-month biomarker assessment, and a 3-month surveillance visit with contrast-enhanced CT of the chest/abdomen/pelvis or pancreas-protocol CT; MRI was used when CT was unsuitable. During the first postoperative year, clinical review was generally performed every 3–4 months and cross-sectional imaging every 3–6 months or earlier when clinically indicated; follow-up was generally every 3–6 months during year 2 and every 6–12 months during years 3–5, with intensity individualized according to clinical status and treatment.

### 2.5 Statistical analysis

Continuous variables were summarized as mean (standard deviation) or median [interquartile range] according to distribution and clinical convention. Categorical variables were summarized as counts and percentages. Clinical, pathologic, treatment, and biomarker characteristics were compared between patients with and without early recurrence using Wilcoxon rank-sum tests for continuous variables and chi-square or Fisher exact tests for categorical variables. Missing measurements were summarized descriptively (Supplementary Table S2 and Supplementary Figure S1). The primary dynamic-pattern analysis used complete data for the required 3-month NLR, CRP, and PNI components; no imputation was used for the composite classification.

Multivariable logistic regression was used to evaluate early recurrence, and Cox proportional hazards models were used for landmark RFS and OS. The primary adjustment set included age per 10-year increase, tumor size, pathologic node-positive disease, lymphovascular invasion, R1 margin, and log2-transformed preoperative CA19-9. Adjuvant chemotherapy was not included in the primary models because treatment began after surgery and overlapped the biomarker ascertainment period. A sensitivity analysis additionally adjusted for adjuvant chemotherapy status as known by POD 105; all documented treatment starts among treated patients occurred by POD 93. Additional sensitivity analyses evaluated the individual dynamic components.

Model performance for early recurrence was assessed using AUC, Brier score, calibration intercept, and calibration slope. Clinical and dynamic models were internally validated using 1,000 bootstrap resamples to estimate optimism-corrected AUC and Brier score and bootstrap calibration parameters; apparent AUCs were compared using DeLong's test. Time-dependent AUCs for RFS and OS were calculated at 12, 24, and 36 months after the landmark. Proportional hazards assumptions were evaluated using Schoenfeld residual tests. For recurrence-specific sensitivity analysis, Fine-Gray regression treated death before documented recurrence as a competing event; for OS, death from any cause was the event and therefore was not treated as a competing risk. Analyses were conducted in R version 4.5.3. All tests were two-sided, and *P* < 0.05 was considered statistically significant. Secondary and sensitivity analyses were considered supportive, and no adjustment for multiple comparisons was applied.

## 3 Results

The screening process is shown in Figure 1. Of 384 patients with pathology-confirmed pancreatic malignancy assessed for surgical treatment, 52 were excluded because of non-PDAC or periampullary non-PDAC pathology, 26 because they underwent palliative bypass or exploration without curative-intent resection, and 6 because of perioperative death before planned biomarker follow-up. A further 15 suspected CA19-9 non-secretors/low expressors and 13 patients with incomplete serial-marker data were excluded from serial-marker eligibility, and 4 patients were excluded because follow-up was shorter than 12 months without recurrence. The full eligible cohort comprised 268 patients. Thirteen patients had unavailable 3-month CRP and were unclassifiable by the primary recovery rule, leaving 255 patients in the primary dynamic-pattern cohort.

**Figure 1:**
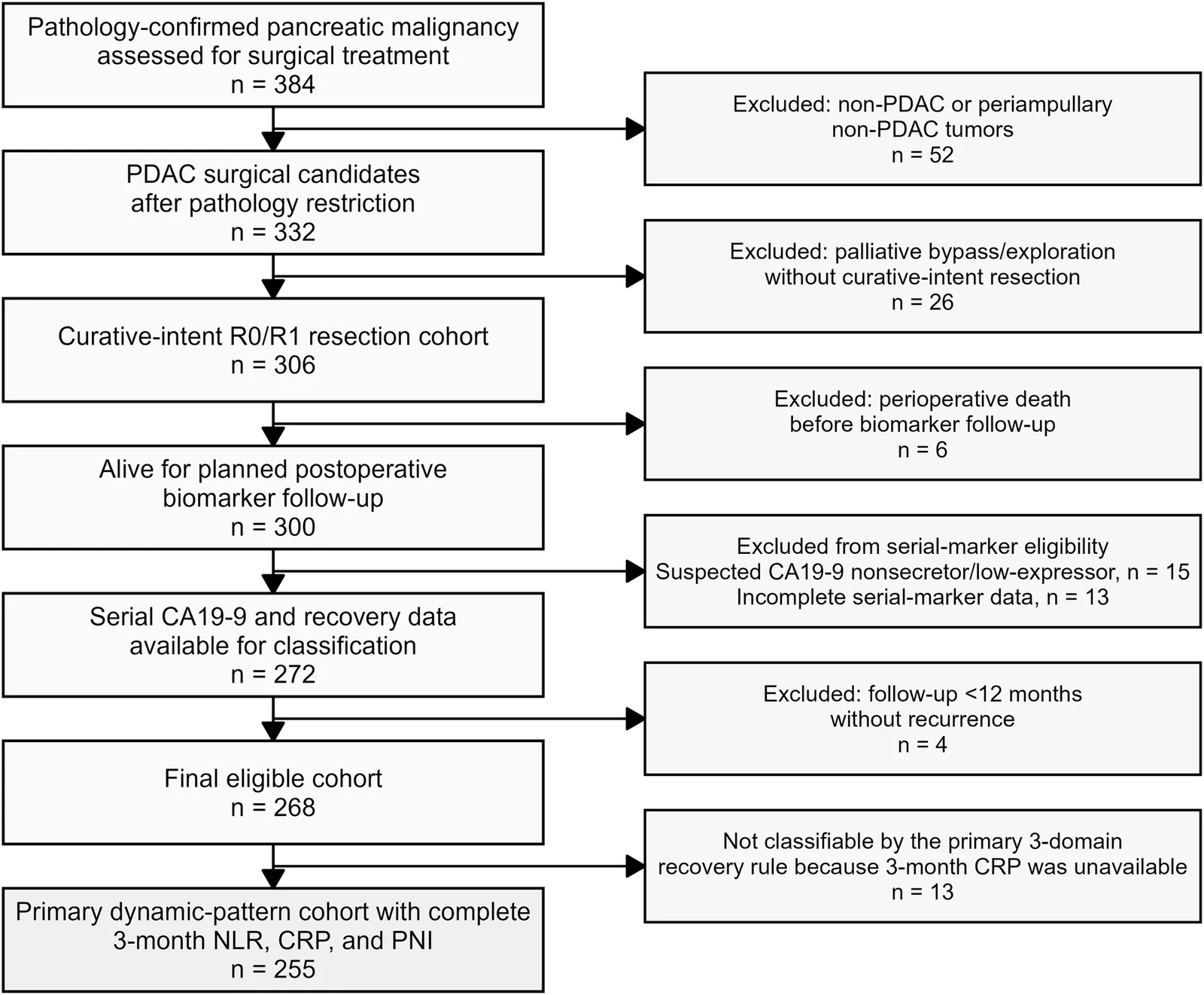
Study cohort screening and analysis flowchart. The full eligible cohort included 268 patients; 255 patients with complete 3-month NLR, CRP, and PNI were classifiable by the primary dynamic-pattern rule. PDAC, pancreatic ductal adenocarcinoma; CRP, C-reactive protein; NLR, neutrophil-to-lymphocyte ratio; PNI, prognostic nutritional index.

Operations were performed between March 5, 2016 and December 31, 2024. The mean age was 64.3 years (SD 8.8), and 142 patients (53.0%) were male. Follow-up was censored on May 31, 2026; the median follow-up time was 32.8 months [IQR, 22.3–50.7]. Early recurrence within 12 months occurred in 78 patients (29.1%). During follow-up, 149 patients (55.6%) developed recurrence and 130 (48.5%) died.

Clinical, pathologic, treatment, and biomarker characteristics stratified by early recurrence are shown in Table 1. Patients with early recurrence had higher median preoperative CA19-9 (250.2 vs. 168.2 U/mL, *P* = 0.008), higher median CA19-9 at 1 month (107.4 vs. 60.8 U/mL, *P* < 0.001), and higher median CA19-9 at 3 months (119.0 vs. 62.5 U/mL, *P* < 0.001). They also had lower preoperative albumin, higher preoperative CRP, more frequent poor histologic grade, more frequent perineural invasion, lower 1-month CA19-9 normalization, and more frequent 3-month CA19-9 rebound. Other perioperative variables, including age, body mass index, tumor location, procedure type, vascular resection, and postoperative complications, were not materially different between groups. Perioperative trajectories of CA19-9, PNI, and NLR stratified by early recurrence status are shown in Figure 2.

**Table 1:** Clinical, pathologic, treatment, and biomarker characteristics stratified by early recurrence.

Characteristic	Overall	No early recurrence	Early recurrence ≤ 12 months	*P* value
Age, years	64.3 (8.8)	63.8 (9.3)	65.4 (7.6)	0.190
Body mass index, kg/m²	22.7 (2.4)	22.6 (2.5)	22.8 (2.3)	0.685
Tumor size, cm	3.2 [2.6, 3.7]	3.2 [2.6, 3.7]	3.1 [2.5, 3.8]	0.575
Lymph nodes examined	17 [13, 21]	17 [13, 21]	18 [13, 22]	0.178
Positive lymph nodes	0 [0, 2]	0 [0, 2]	1 [0, 3]	0.113
Lymph node ratio	0.00 [0.00, 0.12]	0.00 [0.00, 0.12]	0.04 [0.00, 0.12]	0.234
Operation time, min	312 [258, 373]	314 [262, 372]	301 [258, 383]	0.842
Estimated blood loss, mL	455 [270, 650]	450 [270, 668]	470 [275, 640]	0.968
Postoperative length of stay, days	18 [14, 22]	18 [14, 22]	18 [13, 22]	0.780
Preoperative albumin, g/L	37.8 [34.4, 40.8]	38.3 [34.5, 41.4]	36.4 [34.2, 39.4]	0.043
Preoperative CRP, mg/L	7.5 [4.6, 12.6]	7.2 [4.5, 11.2]	9.0 [5.1, 14.7]	0.045
Preoperative NLR	3.62 [2.72, 4.89]	3.51 [2.66, 4.72]	4.07 [2.85, 5.81]	0.073
Preoperative SII	924 [648, 1,278]	922 [623, 1,210]	938 [665, 1,371]	0.112
Preoperative PNI	45.0 [41.2, 48.4]	45.7 [41.3, 49.2]	43.8 [41.0, 47.2]	0.053
Preoperative CA19-9, U/mL	193.8 [74.9, 472.5]	168.2 [66.5, 450.2]	250.2 [130.9, 616.1]	0.008
CA19-9 at 1 month, U/mL	64.8 [37.2, 156.7]	60.8 [32.9, 114.3]	107.4 [50.4, 268.7]	<0.001
CA19-9 at 3 months, U/mL	69.9 [42.0, 160.3]	62.5 [39.7, 106.2]	119.0 [58.1, 279.3]	<0.001
CA19-9 decrease from baseline to 1 month, %	58.7 [38.1, 72.5]	61.2 [38.5, 73.5]	57.7 [37.2, 69.0]	0.648
CA19-9 ratio, 3 months/1 month	1.10 [0.82, 1.44]	1.06 [0.81, 1.37]	1.24 [0.88, 1.69]	0.042
PNI change, 3 months minus baseline	1.8 [−0.2, 3.8]	1.8 [−0.2, 4.1]	1.5 [−0.1, 3.4]	0.402
NLR change, 3 months minus baseline	−0.11 [−0.73, 0.73]	−0.18 [−0.72, 0.67]	0.13 [−0.71, 0.81]	0.217
Sex				0.652
Female	126 (47.0)	91 (47.9)	35 (44.9)	
Male	142 (53.0)	99 (52.1)	43 (55.1)	
ASA class				0.681
ASA 1	54 (20.1)	37 (19.5)	17 (21.8)	
ASA 2	162 (60.4)	118 (62.1)	44 (56.4)	
ASA 3	52 (19.4)	35 (18.4)	17 (21.8)	
Smoking history				0.093
No	188 (70.1)	139 (73.2)	49 (62.8)	
Yes	80 (29.9)	51 (26.8)	29 (37.2)	
Alcohol history				0.574
No	212 (79.1)	152 (80.0)	60 (76.9)	
Yes	56 (20.9)	38 (20.0)	18 (23.1)	
Diabetes				0.162
No	201 (75.0)	147 (77.4)	54 (69.2)	
Yes	67 (25.0)	43 (22.6)	24 (30.8)	
New-onset diabetes				0.269
No	242 (90.3)	174 (91.6)	68 (87.2)	
Yes	26 (9.7)	16 (8.4)	10 (12.8)	
Hypertension				0.803
No	168 (62.7)	120 (63.2)	48 (61.5)	
Yes	100 (37.3)	70 (36.8)	30 (38.5)	
Tumor location				0.821
Head	178 (66.4)	127 (66.8)	51 (65.4)	
Neck	14 (5.2)	9 (4.7)	5 (6.4)	
Body/tail	76 (28.4)	54 (28.4)	22 (28.2)	
Resectability status				0.413
Resectable	208 (77.6)	150 (78.9)	58 (74.4)	
Borderline resectable	60 (22.4)	40 (21.1)	20 (25.6)	
Neoadjuvant therapy				0.208
No	243 (90.7)	175 (92.1)	68 (87.2)	
Yes	25 (9.3)	15 (7.9)	10 (12.8)	
Preoperative jaundice				0.196
No	147 (54.9)	109 (57.4)	38 (48.7)	
Yes	121 (45.1)	81 (42.6)	40 (51.3)	
Preoperative biliary drainage				0.621
No	222 (82.8)	156 (82.1)	66 (84.6)	
Yes	46 (17.2)	34 (17.9)	12 (15.4)	
Procedure				0.889
Pancreaticoduodenectomy	182 (67.9)	129 (67.9)	53 (67.9)	
Distal pancreatectomy	80 (29.9)	56 (29.5)	24 (30.8)	
Total pancreatectomy	6 (2.2)	5 (2.6)	1 (1.3)	
Vascular resection				0.810
No	221 (82.5)	156 (82.1)	65 (83.3)	
Yes	47 (17.5)	34 (17.9)	13 (16.7)	
Pathologic T stage				0.791
T1	22 (8.2)	15 (7.9)	7 (9.0)	
T2	143 (53.4)	105 (55.3)	38 (48.7)	
T3	94 (35.1)	64 (33.7)	30 (38.5)	
T4	9 (3.4)	6 (3.2)	3 (3.8)	
Pathologic N stage				0.053
N0	144 (53.7)	107 (56.3)	37 (47.4)	
N1	100 (37.3)	71 (37.4)	29 (37.2)	
N2	24 (9.0)	12 (6.3)	12 (15.4)	
Pathologic TNM stage				0.116
IA–IB	100 (37.3)	74 (38.9)	26 (33.3)	
IIA	44 (16.4)	33 (17.4)	11 (14.1)	
IIB	100 (37.3)	71 (37.4)	29 (37.2)	
III	24 (9.0)	12 (6.3)	12 (15.4)	
Histologic grade				<0.001
Well	34 (12.7)	23 (12.1)	11 (14.1)	
Moderate	141 (52.6)	115 (60.5)	26 (33.3)	
Poor	93 (34.7)	52 (27.4)	41 (52.6)	
Lymphovascular invasion				0.565
No	155 (57.8)	112 (58.9)	43 (55.1)	
Yes	113 (42.2)	78 (41.1)	35 (44.9)	
Perineural invasion				0.029
No	117 (43.7)	91 (47.9)	26 (33.3)	
Yes	151 (56.3)	99 (52.1)	52 (66.7)	
R1 margin				0.304
No	226 (84.3)	163 (85.8)	63 (80.8)	
Yes	42 (15.7)	27 (14.2)	15 (19.2)	
Clinically relevant POPF				0.972
No	223 (83.2)	158 (83.2)	65 (83.3)	
Yes	45 (16.8)	32 (16.8)	13 (16.7)	
Postoperative infection				0.788
No	209 (78.0)	149 (78.4)	60 (76.9)	
Yes	59 (22.0)	41 (21.6)	18 (23.1)	
Clavien-Dindo grade ≥ III				0.442
No	191 (71.3)	138 (72.6)	53 (67.9)	
Yes	77 (28.7)	52 (27.4)	25 (32.1)	
CA19-9 normalized at 1 month				0.013
No	201 (75.0)	134 (70.5)	67 (85.9)	
Yes	67 (25.0)	56 (29.5)	11 (14.1)	
CA19-9 rebound by 3 months				0.012
No	216 (80.6)	161 (84.7)	55 (70.5)	
Yes	52 (19.4)	29 (15.3)	23 (29.5)	
Inflammatory-nutritional recovery				0.062
Good	60 (22.4)	46 (24.2)	14 (17.9)	
Intermediate	101 (37.7)	78 (41.1)	23 (29.5)	
Poor	94 (35.1)	58 (30.5)	36 (46.2)	
Unclassifiable (3-month CRP unavailable)	13 (4.9)	8 (4.2)	5 (6.4)	
Dynamic recovery pattern				0.005
Favorable	94 (35.1)	75 (39.5)	19 (24.4)	
Intermediate	121 (45.1)	87 (45.8)	34 (43.6)	
Poor	40 (14.9)	20 (10.5)	20 (25.6)	
Unclassifiable (3-month CRP unavailable)	13 (4.9)	8 (4.2)	5 (6.4)	
Adjuvant chemotherapy				0.006
No	39 (14.6)	20 (10.5)	19 (24.4)	
Yes	229 (85.4)	170 (89.5)	59 (75.6)	
Adjuvant chemotherapy timing				0.012
Started within 8 weeks	131 (48.9)	99 (52.1)	32 (41.0)	
Delayed >8 weeks	98 (36.6)	71 (37.4)	27 (34.6)	
No adjuvant	39 (14.6)	20 (10.5)	19 (24.4)	

Values are mean (SD), median [IQR], or *n* (%). *P* values were calculated using Wilcoxon rank-sum tests for continuous variables and chi-square or Fisher exact tests for categorical variables. The recovery and dynamic-pattern rows include an unclassifiable category for 13 patients with unavailable 3-month CRP; primary dynamic-pattern models used the 255 classifiable patients. CA19-9, carbohydrate antigen 19-9; CRP, C-reactive protein; NLR, neutrophil-to-lymphocyte ratio; PNI, prognostic nutritional index; POPF, postoperative pancreatic fistula; SII, systemic immune-inflammation index.

**Figure 2:**
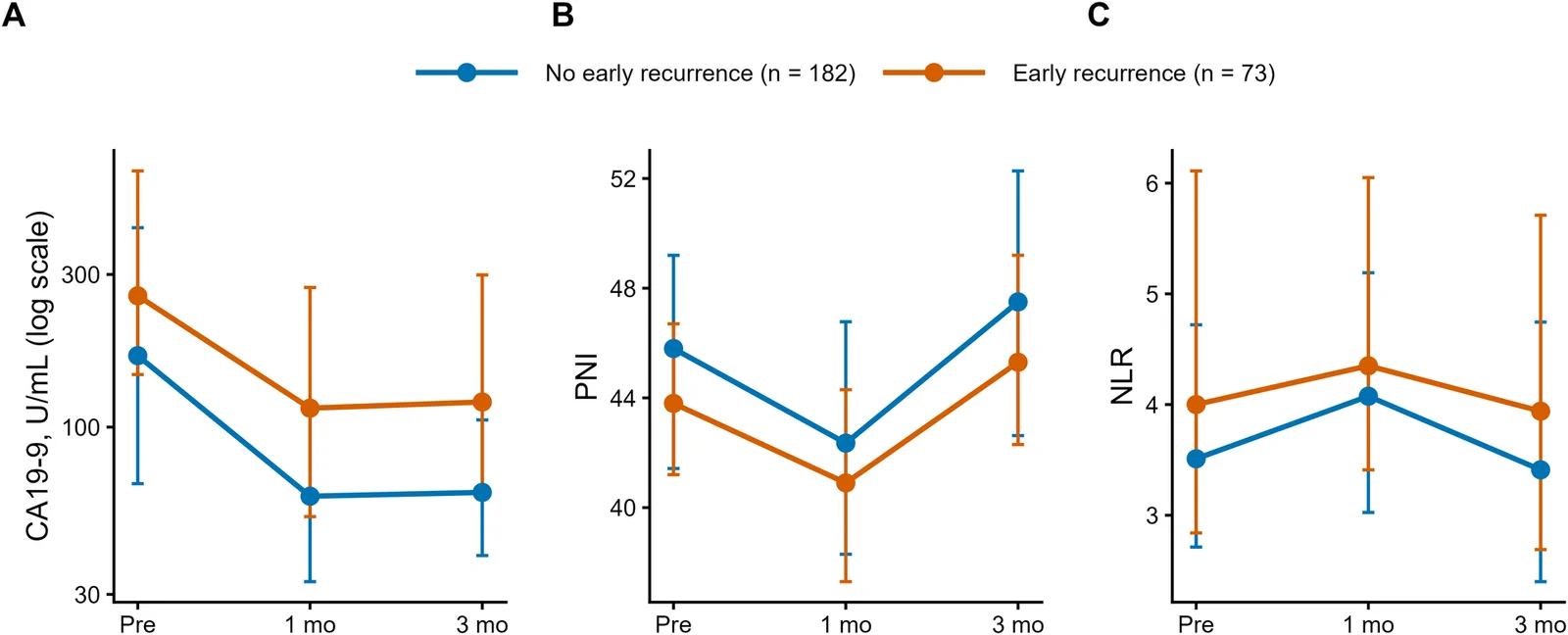
Perioperative trajectories of CA19-9, prognostic nutritional index, and neutrophil-to-lymphocyte ratio in the primary dynamic-pattern cohort, stratified by 12-month early-recurrence status. Points indicate medians and whiskers indicate interquartile ranges. CA19-9 is displayed on a logarithmic scale.

In the primary dynamic-pattern cohort, 94 patients (36.9%) were classified as favorable, 121 (47.5%) as intermediate, and 40 (15.7%) as poor. Early recurrence occurred in 19 of 94 patients (20.2%) in the favorable group, 34 of 121 (28.1%) in the intermediate group, and 20 of 40 (50.0%) in the poor group. Recurrence sites in the full cohort included liver in 38 patients, local recurrence in 36, peritoneum in 31, multiple sites in 30, and lung in 14. The relationships among CA19-9 state, inflammatory-nutritional recovery, the composite dynamic pattern, and 12-month early recurrence are illustrated in Supplementary Figure S3.

The median timing of the 1-month assessment was POD 31 [IQR, 29–34; range, 22–44], and the median timing of 3-month assessment was POD 89 [IQR, 83–94; range, 75–105]. At the 1-month assessment, 216 patients were pre-adjuvant, 13 were on adjuvant therapy, and 39 received no adjuvant therapy; at the 3-month assessment, 212 were on treatment, 16 remained pre-adjuvant, 1 had completed treatment, and 39 received no adjuvant therapy. Among 229 patients who received adjuvant chemotherapy, treatment began at a median POD 54 [IQR, 43–66; range, 25–93], 152 (66.4%) completed the planned course, and the median treatment duration was 153 days [IQR, 112–154; range, 42–160]. Regimen and completion details are provided in Supplementary Table S7, and biomarker timing relative to treatment is summarized in Supplementary Table S8. No recurrence or death occurred before the POD-105 landmark.

Univariable associations are presented in Supplementary Table S3. In the primary multivariable logistic model (Table 2), the adjusted OR for early recurrence was 2.02 (95% CI, 1.01–4.02; *P* = 0.045) for the intermediate pattern and 4.96 (95% CI, 2.10–11.71; *P* < 0.001) for the poor pattern, compared with the favorable pattern. Higher preoperative CA19-9 was also associated with early recurrence (adjusted OR per log2 increase, 1.32; 95% CI, 1.12–1.56; *P* = 0.001). In the sensitivity model additionally adjusted for adjuvant chemotherapy status at POD 105, estimates were 1.99 (95% CI, 1.00–3.99; *P* = 0.052) for the intermediate pattern and 4.30 (95% CI, 1.79–10.33; *P* = 0.001) for the poor pattern (Supplementary Table S11). The continuous association between the 3-month/1-month CA19-9 ratio and early recurrence is shown in Supplementary Figure S2.

**Table 2:** Multivariable logistic regression for early recurrence within 12 months in the primary dynamic-pattern cohort.

Characteristic	Adjusted OR (95% CI)	*P* value
Age, per 10-year increase	1.22 (0.86–1.73)	0.263
Tumor size, per 1 cm increase	0.85 (0.60–1.20)	0.349
Pathologic node-positive disease	1.37 (0.74–2.53)	0.320
Lymphovascular invasion	1.13 (0.61–2.07)	0.700
R1 margin	1.67 (0.78–3.59)	0.186
Preoperative CA19-9, per log2 increase	1.32 (1.12–1.56)	0.001
Dynamic pattern: intermediate vs. favorable	2.02 (1.01–4.02)	0.045
Dynamic pattern: poor vs. favorable	4.96 (2.10–11.71)	<0.001

The favorable dynamic pattern was the reference category. The model included age, tumor size, pathologic node-positive disease, lymphovascular invasion, R1 margin, log2-transformed preoperative CA19-9, and dynamic pattern. Adjuvant chemotherapy was evaluated in a separate sensitivity model (Supplementary Table S11).

OR, odds ratio; CI, confidence interval.

The poor dynamic pattern was also associated with shorter landmark RFS and OS (Table 3 and Figure 3). For RFS, adjusted HRs were 1.40 (95% CI, 0.96–2.05; *P* = 0.084) for the intermediate pattern and 2.12 (95% CI, 1.31–3.42; *P* = 0.002) for the poor pattern. For OS, adjusted HRs were 1.58 (95% CI, 1.02–2.43; *P* = 0.040) and 2.52 (95% CI, 1.50–4.24; *P* < 0.001), respectively. After additional adjustment for adjuvant chemotherapy status at POD 105, the poor pattern remained associated with RFS (HR, 1.73; 95% CI, 1.05–2.86; *P* = 0.032) and OS (HR, 2.08; 95% CI, 1.21–3.57; *P* = 0.008) (Supplementary Table S11). In the recurrence-specific Fine-Gray sensitivity analysis, the poor pattern remained associated with recurrence (sHR, 2.15; 95% CI, 1.28–3.60; *P* = 0.004) (Supplementary Table S10).

**Table 3:** Landmark cox regression for recurrence-free and overall survival in the primary dynamic-pattern cohort.

Outcome	Characteristic	Adjusted HR (95% CI)	*P* value
Recurrence-free survival	Age, per 10-year increase	1.06 (0.89–1.27)	0.524
Recurrence-free survival	Tumor size, per 1 cm increase	0.86 (0.70–1.05)	0.135
Recurrence-free survival	Pathologic node-positive disease	1.27 (0.89–1.82)	0.186
Recurrence-free survival	Lymphovascular invasion	1.00 (0.70–1.42)	0.986
Recurrence-free survival	R1 margin	1.46 (0.94–2.28)	0.095
Recurrence-free survival	Preoperative CA19-9, per log2 increase	1.12 (1.02–1.23)	0.016
Recurrence-free survival	Dynamic pattern: intermediate vs. favorable	1.40 (0.96–2.05)	0.084
Recurrence-free survival	Dynamic pattern: poor vs. favorable	2.12 (1.31–3.42)	0.002
Overall survival	Age, per 10-year increase	1.10 (0.90–1.35)	0.336
Overall survival	Tumor size, per 1 cm increase	0.90 (0.73–1.12)	0.353
Overall survival	Pathologic node-positive disease	1.33 (0.89–1.98)	0.164
Overall survival	Lymphovascular invasion	1.01 (0.68–1.50)	0.951
Overall survival	R1 margin	1.64 (1.01–2.66)	0.045
Overall survival	Preoperative CA19-9, per log2 increase	1.14 (1.03–1.27)	0.010
Overall survival	Dynamic pattern: intermediate vs. favorable	1.58 (1.02–2.43)	0.040
Overall survival	Dynamic pattern: poor vs. favorable	2.52 (1.50–4.24)	<0.001

Follow-up began at the POD-105 landmark. RFS was defined by recurrence or death from any cause; OS was defined by all-cause death. The favorable dynamic pattern was the reference category. Models used the same adjustment set as Table 2. Adjuvant chemotherapy was evaluated in a separate sensitivity model (Supplementary Table S11).

HR, hazard ratio; CI, confidence interval.

**Figure 3:**
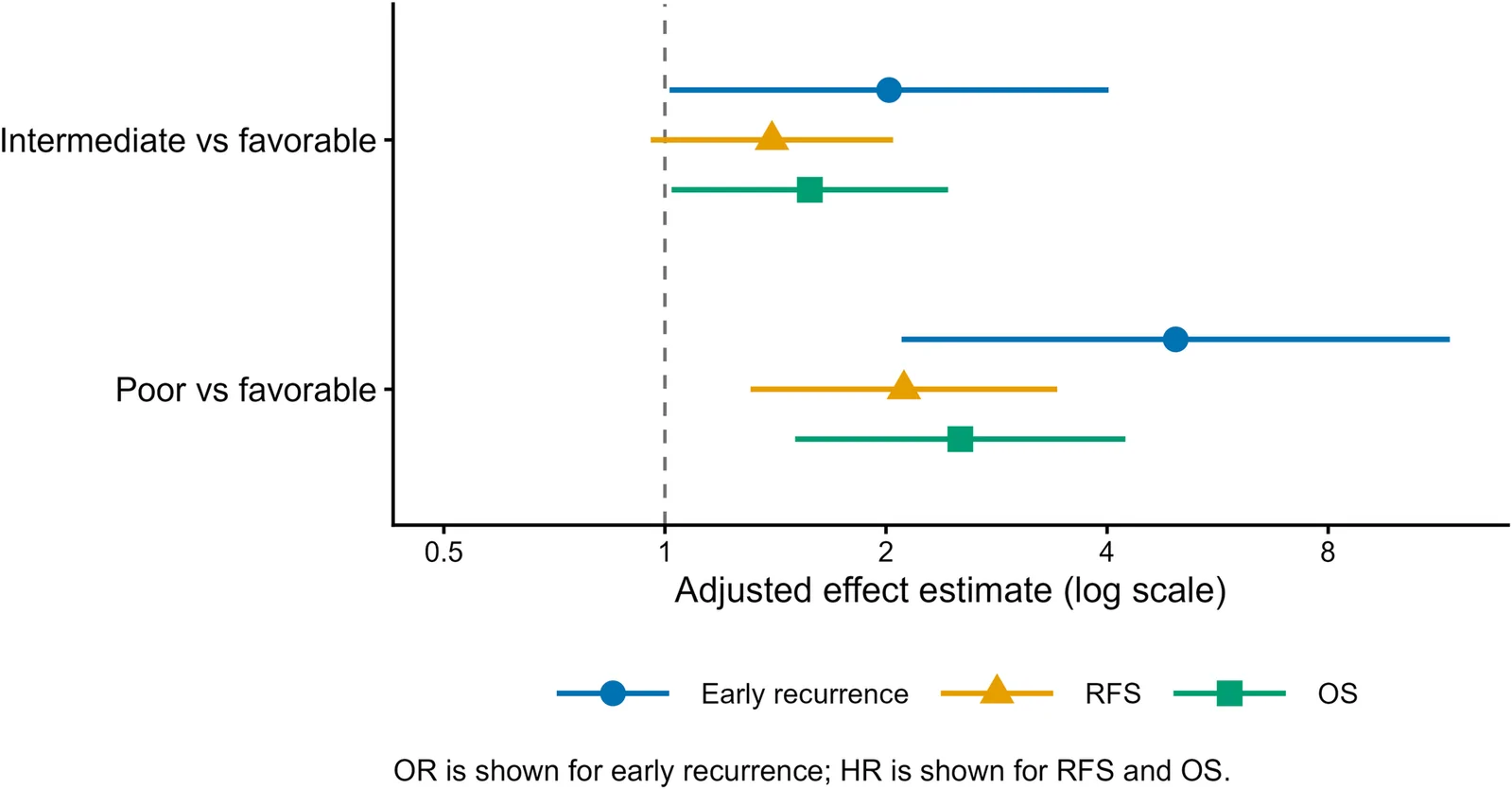
Adjusted associations of the dynamic recovery pattern with early recurrence, landmark recurrence-free survival, and landmark overall survival. The favorable pattern is the reference category. ORs are shown for early recurrence and HRs for RFS and OS; horizontal bars indicate 95% CIs. Models were adjusted for age, tumor size, pathologic node-positive disease, lymphovascular invasion, R1 margin, and log2-transformed preoperative CA19-9.

Kaplan–Meier curves separated across dynamic recovery groups after the POD-105 landmark (Figure 4). Median RFS was 37.9 months (95% CI lower bound, 27.8 months; upper bound not estimable) in the favorable group, 27.5 months (95% CI, 21.1–36.4) in the intermediate group, and 10.0 months (95% CI, 5.4–26.5) in the poor group (log-rank *P* = 0.010). Median OS was 47.8 months (95% CI lower bound, 43.4 months; upper bound not estimable), 46.1 months (95% CI, 41.3–53.2), and 29.0 months (95% CI, 20.3–45.5), respectively (log-rank *P* = 0.003).

**Figure 4:**
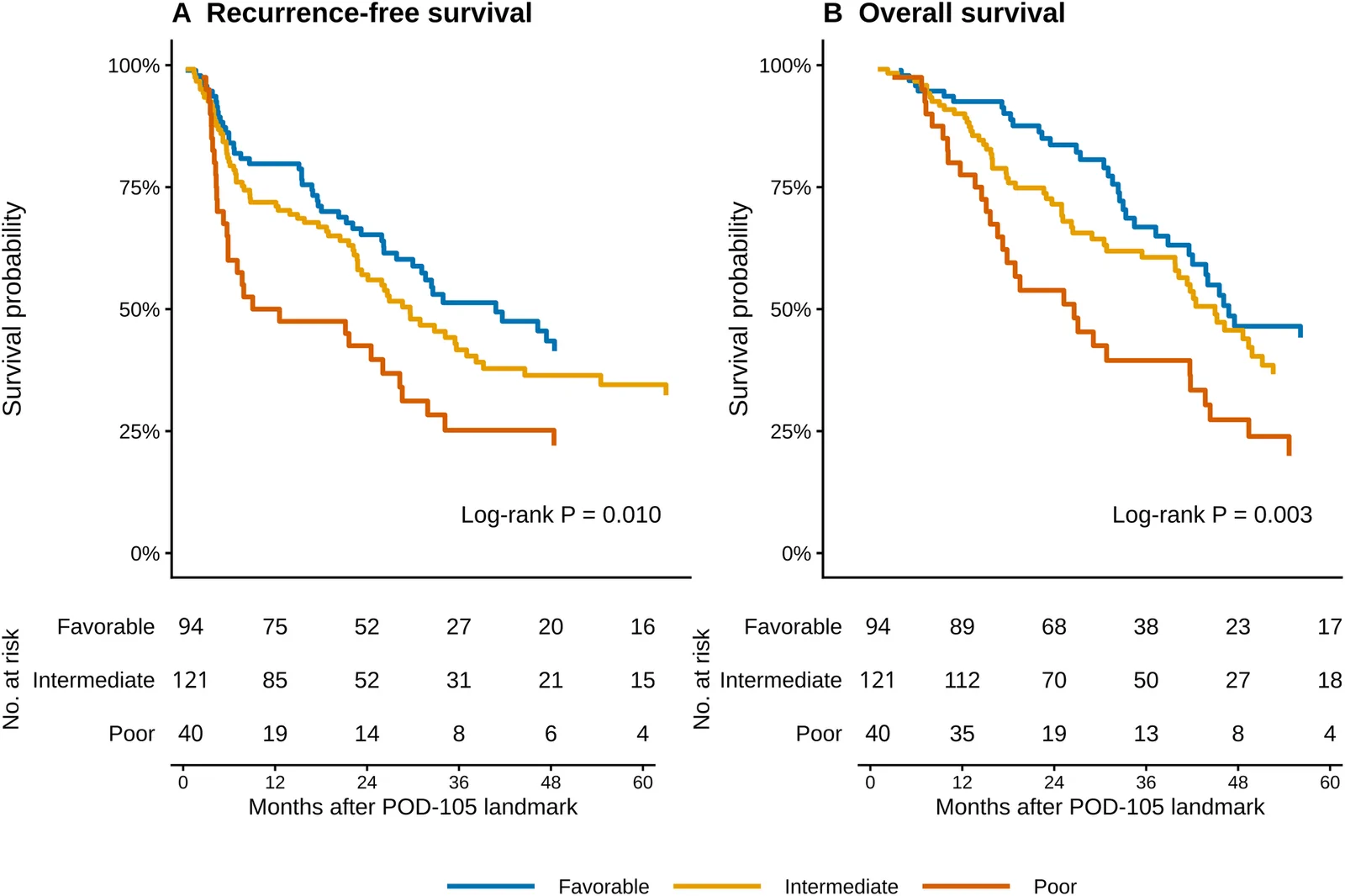
Kaplan–meier curves for recurrence-free survival and overall survival from the postoperative day-105 landmark according to dynamic recovery pattern. Risk tables show the number of patients at risk at selected time points after the landmark. RFS, recurrence-free survival; OS, overall survival.

Performance results are summarized in Table 4 and Figure 5. In the 255-patient primary cohort, the clinical model had an apparent AUC of 0.648 and the model adding the dynamic pattern had an apparent AUC of 0.702; the difference did not reach conventional statistical significance (DeLong *P* = 0.057). Optimism-corrected AUCs from 1,000 bootstrap resamples were 0.605 and 0.661, respectively. Corresponding optimism-corrected Brier scores were 0.204 and 0.194, and bootstrap calibration slopes were 0.749 and 0.788 (Supplementary Table S9). Individual-component sensitivity analyses showed associations of 3-month CA19-9 rebound and poor recovery with early recurrence, while poor recovery remained associated with RFS and OS (Supplementary Table S4). Landmark time-dependent AUCs are reported in Supplementary Table S5, and proportional hazards tests showed no evidence of a global violation for RFS or OS (Supplementary Table S6).

**Table 4:** Early-recurrence model performance and bootstrap internal validation.

Model	Apparent AUC	Optimism-corrected AUC	Apparent brier	Optimism-corrected brier	Bootstrap calibration intercept	Bootstrap calibration slope
Clinical	0.648	0.605	0.192	0.204	−0.212	0.749
Clinical + dynamic pattern	0.702	0.661	0.181	0.194	−0.168	0.788

Both models were evaluated in the same 255-patient primary cohort. The clinical model included age, tumor size, pathologic node-positive disease, lymphovascular invasion, R1 margin, and log2-transformed preoperative CA19-9; the dynamic model additionally included the three-level dynamic pattern. Bootstrap estimates used 1,000 resamples. The apparent AUC difference was assessed using DeLong's test (*P* = 0.057).

AUC, area under the receiver operating characteristic curve.

**Figure 5:**
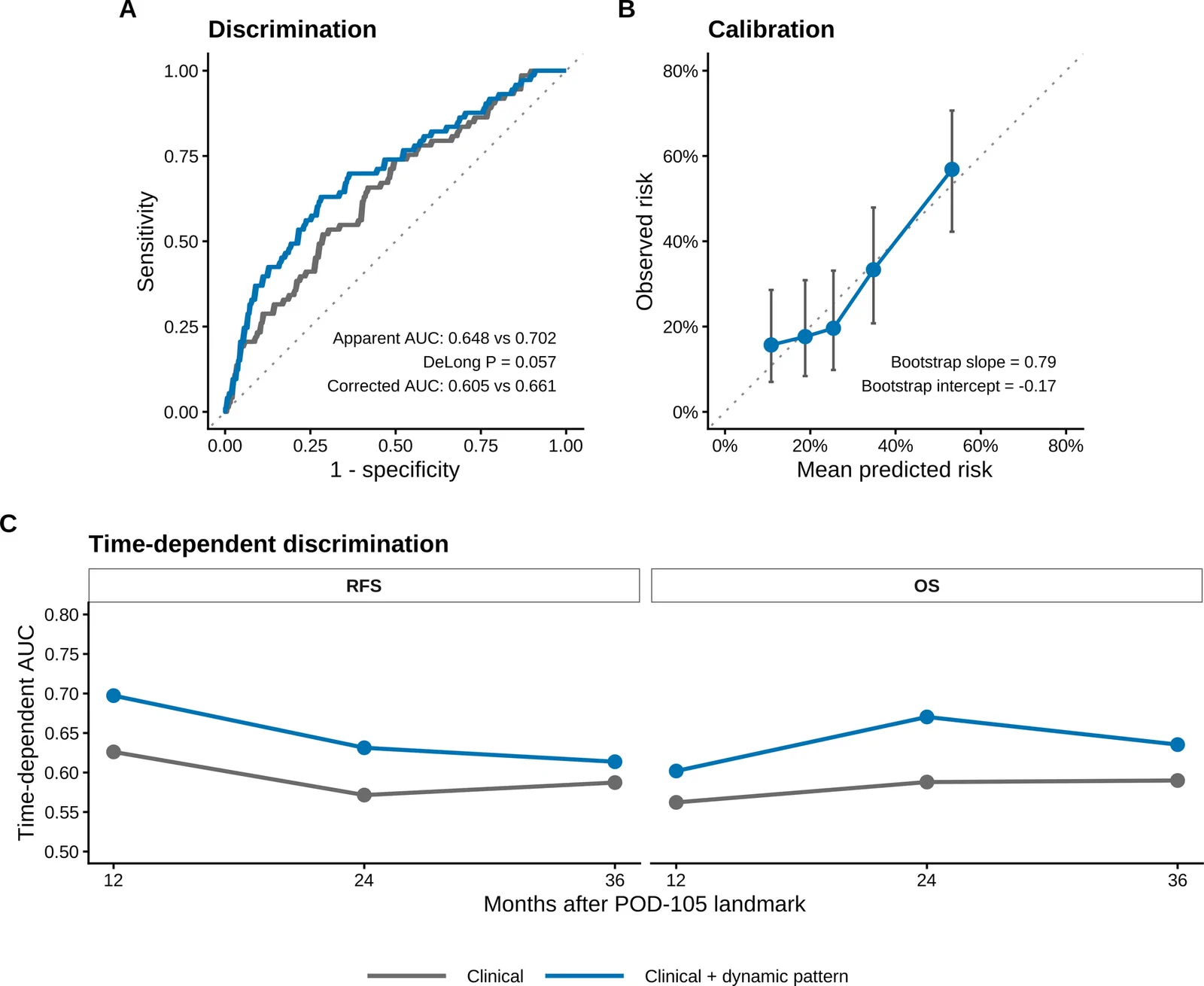
Performance of the early-recurrence risk models. Panel A shows receiver operating characteristic curves for the clinical model and the model adding the dynamic recovery pattern, with apparent and optimism-corrected AUCs. Panel B shows grouped apparent calibration of the dynamic model and bootstrap calibration parameters. Panel C shows landmark time-dependent AUCs for RFS and OS at 12, 24, and 36 months. AUC, area under the receiver operating characteristic curve.

## 4 Discussion

This study found that a transparent three-level postoperative dynamic pattern integrating CA19-9 behavior with inflammatory-nutritional recovery was associated with early recurrence and survival after curative-intent resection for PDAC. The association was most consistent for the poor pattern, which had an adjusted OR of 4.96 for early recurrence, an adjusted HR of 2.12 for RFS, and an adjusted HR of 2.52 for OS compared with the favorable pattern. Associations for the intermediate pattern were weaker and less consistent across endpoints and sensitivity analyses. These findings support a postoperative prognostic signal rather than a stand-alone clinical prediction rule.

The recurrence rate observed here is consistent with the clinical reality that PDAC often behaves as a systemic disease even when resection is technically feasible. Groot and colleagues showed that recurrence after pancreatectomy follows distinct temporal and anatomical patterns, and early recurrence is a marker of aggressive disease biology (13). In a large resected cohort, early recurrence could be predicted but remained difficult to capture using conventional clinicopathologic factors alone (11). A recent systematic review and meta-analysis also confirmed that early recurrence after pancreatic cancer resection is influenced by multiple tumor- and treatment-related factors rather than a single dominant predictor (12). Our data fit this framework because the dynamic pattern summarized postoperative biologic behavior and host recovery rather than simply restating baseline tumor stage.

CA19-9 remains central to PDAC management, but its interpretation is strongest when placed in clinical context. Montgomery et al. reported that post-resection CA19-9 values were associated with recurrence and survival nearly three decades ago (15). Ferrone et al. later showed that perioperative CA19-9 levels could predict stage and survival in patients with resectable pancreatic adenocarcinoma (16). These findings have been extended by postoperative surveillance studies showing that CA19-9 trends can alter recurrence probability over time (17). The present study supports that longitudinal interpretation: postoperative CA19-9 behavior contributed prognostic information when interpreted together with recovery markers, but the incremental discrimination beyond clinicopathologic variables was modest rather than clearly superior.

The emphasis on normalization and rebound is also consistent with recent multimodal-treatment literature. DiPeri et al. found that preoperative and postoperative CA19-9 normalization carried prognostic information in patients treated with neoadjuvant therapy or surgery first (18). Newhook et al. showed that CA19-9 response dynamics and normalization during neoadjuvant therapy were associated with prognosis after resection (19). Our cohort differs in that the dynamic pattern was anchored to early postoperative recovery rather than neoadjuvant response alone. Importantly, most 3-month samples were obtained while patients were receiving adjuvant chemotherapy, so the 3-month component should be interpreted as a treatment-exposed postoperative state rather than an untreated baseline measurement.

At the same time, CA19-9 is not a perfect marker. Its serum level can be affected by biliary obstruction, inflammation, and assay context, and a subset of patients with Lewis antigen-related non-secretor status may not produce informative CA19-9 levels (20). Biochemical reviews have also emphasized that CA19-9 should not be interpreted as a tumor-specific marker in isolation (21). In the present cohort, 15 patients were excluded as suspected CA19-9 non-secretors/low expressors, whereas 13 additional patients were excluded because serial-marker data were incomplete. Serial postoperative CA125 and CEA measurements were not available with sufficient consistency to support a validated substitution algorithm. Accordingly, the current dynamic pattern applies only to patients with informative serial CA19-9, and alternative-marker strategies require separate validation.

The inflammatory-nutritional component is biologically plausible. Systemic inflammation and poor nutritional status are linked to cancer survival through mechanisms involving catabolism, immune dysfunction, treatment intolerance, and impaired recovery (22). In pancreatic cancer, NLR has repeatedly been associated with prognosis, including in a cohort of operable and inoperable patients and in meta-analyses of resected populations (23, 24, 30). PNI has also been linked to prognosis in pancreatic cancer, with meta-analyses supporting its role in patients undergoing curative resection (25, 26). SII provides a related measure of immune-inflammatory balance, and meta-analyses have reported its prognostic value in pancreatic cancer (27, 31). Our results do not establish superiority of one inflammatory marker or a biological interaction among markers; rather, they suggest that a simple recovery classification across complementary inflammatory and nutritional domains may provide clinically interpretable prognostic information.

The survival analyses reinforce the early-recurrence findings, particularly for the poor pattern. The intermediate group showed a smaller and less stable association: its RFS estimate did not reach statistical significance, and the OS association attenuated after additional adjustment for adjuvant therapy status. This heterogeneity argues against treating the intermediate category as uniformly high risk. Instead, the three-level pattern appears most useful for identifying a clearly adverse poor-recovery group while preserving uncertainty in patients with discordant signals.

Adjuvant chemotherapy was examined as a sensitivity covariate rather than as a surgery-time baseline factor. Among treated patients, all documented treatment starts occurred by POD 93, before the POD-105 landmark. Additional adjustment for adjuvant status at the landmark attenuated some estimates but the poor dynamic pattern remained associated with early recurrence, RFS, and OS. These treatment associations should not be interpreted as randomized treatment effects, because real-world treatment selection is influenced by fitness, postoperative recovery, physician judgment, and patient preference. Randomized evidence nevertheless supports adjuvant systemic therapy after PDAC resection; CONKO-001 established the value of adjuvant gemcitabine, ESPAC-4 showed benefit with gemcitabine plus capecitabine, and PRODIGE 24/CCTG PA.6 demonstrated superior outcomes with modified FOLFIRINOX in selected fit patients (6–8). The present analysis therefore treats adjuvant therapy as an important treatment context and sensitivity factor, not as a causal exposure whose effect can be estimated from this observational cohort.

From a modelling perspective, adding the dynamic pattern increased the apparent early-recurrence AUC from 0.648 to 0.702, but the difference did not reach conventional statistical significance (DeLong *P* = 0.057). After 1,000 bootstrap resamples, optimism-corrected AUCs were 0.605 for the clinical model and 0.661 for the dynamic model, with calibration slopes of 0.749 and 0.788, respectively. These estimates indicate modest incremental discrimination with measurable optimism rather than predictive superiority. TRIPOD emphasizes transparent reporting and validation before prediction models are used for individual decision-making (29). Accordingly, the current findings are best viewed as a risk-stratification signal requiring independent external validation, not as evidence for immediate clinical deployment.

The study has limitations. First, it was retrospective and single-center, so selection bias, local treatment patterns, and surveillance practice may influence the findings; treatment and surveillance practices may also have changed over the 2016–2024 surgical period, and residual confounding by other pathologic characteristics cannot be excluded. Second, the dynamic approach is not applicable to all resected PDAC patients: 15 suspected CA19-9 non-secretors/low expressors and 13 patients with incomplete serial-marker data were excluded from serial-marker eligibility, and a further 13 patients lacked 3-month CRP required for the three-domain recovery definition. Third, the operational NLR, CRP, and PNI thresholds used to define recovery should not be regarded as established clinical cut points and require validation in independent cohorts. Fourth, most 3-month measurements were obtained during adjuvant chemotherapy, so the 3-month pattern reflects both postoperative recovery and treatment exposure. Finally, internal validation showed only moderate optimism-corrected discrimination, and no external validation was available. These limitations preclude interpretation of the pattern as a validated clinical decision tool.

Despite these limitations, the study addresses a practical gap by evaluating information that becomes available during early postoperative follow-up rather than relying only on pathology and preoperative markers. The poor dynamic pattern identified a subgroup with consistently adverse outcomes across the primary and sensitivity analyses. If the operational definition is reproduced and validated in independent cohorts, it may provide additional context for postoperative risk assessment and the timing of reassessment, while surveillance and treatment decisions should continue to follow established clinical criteria.

## 5 Conclusion

In this real-world cohort of patients undergoing curative-intent resection for PDAC, a poor postoperative dynamic pattern integrating CA19-9 behavior and inflammatory-nutritional recovery was consistently associated with early recurrence, RFS, and OS. Adding the dynamic pattern provided modest incremental discrimination beyond clinicopathologic variables, but performance remained moderate after bootstrap correction. Independent external validation of the operational thresholds and classification is required before routine clinical use.

## Data Availability

The original contributions presented in the study are included in the article/Supplementary Material, further inquiries can be directed to the corresponding author.
