# Supplementary material for: Dynamic perioperative CA19-9 and inflammatory-nutritional recovery patterns are associated with early recurrence and survival after resection for pancreatic ductal adenocarcinoma: a real-world cohort study
Source: Front Med (Lausanne). 2026 Sep 16;13:1899908. doi: 10.3389/fmed.2026.1899908 (PMC13623743; doi:10.3389/fmed.2026.1899908)
Supplement: Supplementary file 1 [file Table1.docx]

**Supplementary Materials**

*Dynamic perioperative CA19-9 and inflammatory-nutritional recovery patterns are associated with early recurrence and survival after resection for pancreatic ductal adenocarcinoma: a real-world cohort study*

# Supplementary Tables

**Supplementary Table S1. Operational definitions of key variables and outcomes.**

| **Variable** | **Operational definition** |
| --- | --- |
| Early recurrence | Documented recurrence occurring after the POD-105 landmark and within 365 days after surgery. |
| RFS | Time from the POD-105 landmark to first recurrence or death from any cause; patients without an event were censored at last follow-up. |
| OS | Time from the POD-105 landmark to death from any cause; survivors were censored at last follow-up. |
| 1-month CA19-9 normalization | CA19-9 <=37 U/mL at the 1-month postoperative assessment. |
| Marked CA19-9 decline | At least 50% decrease in CA19-9 from the preoperative value to the 1-month assessment. |
| 3-month CA19-9 rebound | A 3-month/1-month CA19-9 ratio >=1.50 together with a 3-month CA19-9 concentration >37 U/mL. |
| Favorable CA19-9 state | 1-month normalization or >=50% decline without a 3-month rebound. |
| Adverse CA19-9 state | All CA19-9 combinations not meeting the favorable-state definition. |
| Good recovery | All three 3-month criteria met: NLR <=5, CRP <=10 mg/L, and PNI >=45. |
| Intermediate recovery | Exactly two of the three 3-month recovery criteria met. |
| Poor recovery | Zero or one of the three 3-month recovery criteria met. |
| Dynamic-pattern classifiability | Complete 3-month NLR, CRP, and PNI were required; patients missing any recovery domain were not assigned a dynamic pattern. |
| Favorable dynamic pattern | Favorable CA19-9 state together with good or intermediate recovery. |
| Intermediate dynamic pattern | All remaining classifiable discordant combinations. |
| Poor dynamic pattern | Adverse CA19-9 state together with poor recovery. |
| Competing-risk recurrence endpoint | Recurrence was the event of interest; death before documented recurrence was treated as a competing event. |

**Supplementary Table S2. Missing laboratory measurements in the full eligible cohort.**

| **Variable** | **Missing, n** | **Missing, %** |
| --- | --- | --- |
| Preoperative CA125, U/mL | 19 | 7.1% |
| CRP at 1 month, mg/L | 16 | 6.0% |
| CRP at 3 months, mg/L | 13 | 4.9% |
| Preoperative CEA, ng/mL | 13 | 4.9% |
| Preoperative total bilirubin, umol/L | 6 | 2.2% |
| Preoperative CRP, mg/L | 5 | 1.9% |
| Preoperative direct bilirubin, umol/L | 5 | 1.9% |

Structurally non-applicable fields, such as recurrence date in patients without recurrence and chemotherapy fields in untreated patients, were not classified as missing data.

**Supplementary Table S3. Univariable associations with early recurrence, landmark recurrence-free survival, and landmark overall survival.**

| **Outcome** | **N** | **Characteristic** | **Effect estimate (95% CI)** | **P value** |
| --- | --- | --- | --- | --- |
| Early recurrence within 12 months | 255 | Age, per 10-year increase | 1.21 (0.88-1.66) | 0.235 |
| Early recurrence within 12 months | 255 | Tumor size, per 1-cm increase | 0.93 (0.67-1.28) | 0.645 |
| Early recurrence within 12 months | 255 | Pathologic node-positive disease | 1.63 (0.95-2.82) | 0.079 |
| Early recurrence within 12 months | 255 | Lymphovascular invasion | 1.30 (0.75-2.25) | 0.345 |
| Early recurrence within 12 months | 255 | R1 margin | 1.49 (0.73-3.06) | 0.277 |
| Early recurrence within 12 months | 255 | Preoperative CA19-9, per log2 increase | 1.24 (1.07-1.44) | 0.004 |
| Early recurrence within 12 months | 255 | CA19-9 normalized at 1 month | 0.38 (0.18-0.79) | 0.010 |
| Early recurrence within 12 months | 255 | CA19-9 rebound by 3 months | 2.28 (1.20-4.31) | 0.012 |
| Early recurrence within 12 months | 255 | Recovery: intermediate vs good | 0.97 (0.45-2.07) | 0.935 |
| Early recurrence within 12 months | 255 | Recovery: poor vs good | 2.04 (0.98-4.23) | 0.055 |
| Early recurrence within 12 months | 255 | Dynamic pattern: intermediate vs favorable | 1.54 (0.81-2.93) | 0.185 |
| Early recurrence within 12 months | 255 | Dynamic pattern: poor vs favorable | 3.95 (1.78-8.77) | <0.001 |
| RFS from POD-105 landmark | 255 | Age, per 10-year increase | 1.05 (0.88-1.25) | 0.569 |
| RFS from POD-105 landmark | 255 | Tumor size, per 1-cm increase | 0.91 (0.75-1.11) | 0.344 |
| RFS from POD-105 landmark | 255 | Pathologic node-positive disease | 1.36 (0.98-1.89) | 0.062 |
| RFS from POD-105 landmark | 255 | Lymphovascular invasion | 1.15 (0.83-1.60) | 0.390 |
| RFS from POD-105 landmark | 255 | R1 margin | 1.35 (0.88-2.09) | 0.168 |
| RFS from POD-105 landmark | 255 | Preoperative CA19-9, per log2 increase | 1.09 (1.00-1.19) | 0.043 |
| RFS from POD-105 landmark | 255 | CA19-9 normalized at 1 month | 0.70 (0.47-1.05) | 0.084 |
| RFS from POD-105 landmark | 255 | CA19-9 rebound by 3 months | 1.22 (0.82-1.82) | 0.321 |
| RFS from POD-105 landmark | 255 | Recovery: intermediate vs good | 1.02 (0.65-1.60) | 0.944 |
| RFS from POD-105 landmark | 255 | Recovery: poor vs good | 1.70 (1.10-2.63) | 0.016 |
| RFS from POD-105 landmark | 255 | Dynamic pattern: intermediate vs favorable | 1.28 (0.88-1.86) | 0.190 |
| RFS from POD-105 landmark | 255 | Dynamic pattern: poor vs favorable | 2.02 (1.28-3.20) | 0.003 |
| OS from POD-105 landmark | 255 | Age, per 10-year increase | 1.10 (0.91-1.34) | 0.328 |
| OS from POD-105 landmark | 255 | Tumor size, per 1-cm increase | 0.99 (0.80-1.22) | 0.894 |
| OS from POD-105 landmark | 255 | Pathologic node-positive disease | 1.45 (1.02-2.08) | 0.040 |
| OS from POD-105 landmark | 255 | Lymphovascular invasion | 1.20 (0.84-1.71) | 0.326 |
| OS from POD-105 landmark | 255 | R1 margin | 1.44 (0.91-2.29) | 0.122 |
| OS from POD-105 landmark | 255 | Preoperative CA19-9, per log2 increase | 1.11 (1.01-1.21) | 0.037 |
| OS from POD-105 landmark | 255 | CA19-9 normalized at 1 month | 0.67 (0.43-1.05) | 0.081 |
| OS from POD-105 landmark | 255 | CA19-9 rebound by 3 months | 1.26 (0.81-1.94) | 0.302 |
| OS from POD-105 landmark | 255 | Recovery: intermediate vs good | 1.07 (0.65-1.77) | 0.798 |
| OS from POD-105 landmark | 255 | Recovery: poor vs good | 1.94 (1.21-3.10) | 0.006 |
| OS from POD-105 landmark | 255 | Dynamic pattern: intermediate vs favorable | 1.38 (0.91-2.10) | 0.128 |
| OS from POD-105 landmark | 255 | Dynamic pattern: poor vs favorable | 2.31 (1.42-3.77) | <0.001 |

Effect estimates are odds ratios for early recurrence and hazard ratios for RFS and OS. RFS and OS follow-up begins at POD 105.

**Supplementary Table S4. Sensitivity analysis using individual postoperative dynamic components.**

| **Outcome** | **Characteristic** | **Adjusted effect estimate (95% CI)** | **P value** |
| --- | --- | --- | --- |
| Early recurrence | Age, per 10-year increase | 1.23 (0.87-1.75) | 0.234 |
| Early recurrence | Tumor size, per 1-cm increase | 0.87 (0.62-1.24) | 0.444 |
| Early recurrence | Pathologic node-positive disease | 1.38 (0.74-2.55) | 0.311 |
| Early recurrence | Lymphovascular invasion | 1.11 (0.60-2.05) | 0.734 |
| Early recurrence | R1 margin | 1.52 (0.70-3.31) | 0.289 |
| Early recurrence | Preoperative CA19-9, per log2 increase | 1.18 (0.98-1.43) | 0.077 |
| Early recurrence | CA19-9 normalized at 1 month | 0.47 (0.19-1.17) | 0.106 |
| Early recurrence | CA19-9 rebound by 3 months | 2.34 (1.17-4.69) | 0.017 |
| Early recurrence | Recovery: intermediate vs good | 1.08 (0.49-2.39) | 0.849 |
| Early recurrence | Recovery: poor vs good | 2.32 (1.06-5.06) | 0.035 |
| RFS | Age, per 10-year increase | 1.05 (0.87-1.25) | 0.614 |
| RFS | Tumor size, per 1-cm increase | 0.86 (0.71-1.05) | 0.148 |
| RFS | Pathologic node-positive disease | 1.26 (0.88-1.79) | 0.210 |
| RFS | Lymphovascular invasion | 1.02 (0.72-1.44) | 0.926 |
| RFS | R1 margin | 1.46 (0.94-2.27) | 0.094 |
| RFS | Preoperative CA19-9, per log2 increase | 1.07 (0.96-1.19) | 0.209 |
| RFS | CA19-9 normalized at 1 month | 0.80 (0.50-1.28) | 0.346 |
| RFS | CA19-9 rebound by 3 months | 1.17 (0.78-1.76) | 0.451 |
| RFS | Recovery: intermediate vs good | 1.04 (0.66-1.64) | 0.860 |
| RFS | Recovery: poor vs good | 1.71 (1.10-2.66) | 0.017 |
| OS | Age, per 10-year increase | 1.09 (0.89-1.33) | 0.417 |
| OS | Tumor size, per 1-cm increase | 0.90 (0.73-1.11) | 0.332 |
| OS | Pathologic node-positive disease | 1.34 (0.90-1.98) | 0.152 |
| OS | Lymphovascular invasion | 1.03 (0.70-1.53) | 0.871 |
| OS | R1 margin | 1.64 (1.02-2.64) | 0.042 |
| OS | Preoperative CA19-9, per log2 increase | 1.08 (0.96-1.21) | 0.220 |
| OS | CA19-9 normalized at 1 month | 0.73 (0.43-1.24) | 0.241 |
| OS | CA19-9 rebound by 3 months | 1.16 (0.74-1.82) | 0.504 |
| OS | Recovery: intermediate vs good | 1.12 (0.67-1.86) | 0.662 |
| OS | Recovery: poor vs good | 2.00 (1.23-3.24) | 0.005 |

Odds ratios are reported for early recurrence and hazard ratios for RFS and OS. Models used the same clinicopathologic adjustment set as the primary analyses.

**Supplementary Table S5. Time-dependent AUCs after the POD-105 landmark.**

| **Outcome** | **Model** | **Months after landmark** | **AUC** |
| --- | --- | --- | --- |
| RFS | Clinical | 12 | 0.626 |
| RFS | Clinical | 24 | 0.571 |
| RFS | Clinical | 36 | 0.587 |
| RFS | Clinical + dynamic pattern | 12 | 0.697 |
| RFS | Clinical + dynamic pattern | 24 | 0.631 |
| RFS | Clinical + dynamic pattern | 36 | 0.614 |
| OS | Clinical | 12 | 0.562 |
| OS | Clinical | 24 | 0.588 |
| OS | Clinical | 36 | 0.590 |
| OS | Clinical + dynamic pattern | 12 | 0.602 |
| OS | Clinical + dynamic pattern | 24 | 0.670 |
| OS | Clinical + dynamic pattern | 36 | 0.635 |

**Supplementary Table S6. Proportional hazards assumption tests for the primary landmark Cox models.**

| **Outcome** | **Characteristic** | **Chi-square** | **df** | **P value** |
| --- | --- | --- | --- | --- |
| RFS | Age, per 10-year increase | 0.76 | 1 | 0.382 |
| RFS | Tumor size, per 1-cm increase | 0.07 | 1 | 0.798 |
| RFS | Pathologic node-positive disease | 1.28 | 1 | 0.257 |
| RFS | Lymphovascular invasion | 0.07 | 1 | 0.797 |
| RFS | R1 margin | 0.04 | 1 | 0.845 |
| RFS | Preoperative CA19-9, per log2 increase | 2.14 | 1 | 0.144 |
| RFS | Dynamic recovery pattern | 1.95 | 2 | 0.378 |
| RFS | GLOBAL | 8.41 | 8 | 0.394 |
| OS | Age, per 10-year increase | 0.76 | 1 | 0.383 |
| OS | Tumor size, per 1-cm increase | 0.73 | 1 | 0.392 |
| OS | Pathologic node-positive disease | 0.94 | 1 | 0.333 |
| OS | Lymphovascular invasion | 0.20 | 1 | 0.656 |
| OS | R1 margin | 0.74 | 1 | 0.391 |
| OS | Preoperative CA19-9, per log2 increase | 0.07 | 1 | 0.791 |
| OS | Dynamic recovery pattern | 3.29 | 2 | 0.193 |
| OS | GLOBAL | 8.49 | 8 | 0.387 |

**Supplementary Table S7. Adjuvant chemotherapy timing, regimen, duration, and completion.**

| **Domain** | **Characteristic** | **Value** |
| --- | --- | --- |
| Treatment exposure | Received adjuvant chemotherapy | 229 (85.4%) |
| Treatment exposure | No adjuvant chemotherapy | 39 (14.6%) |
| Treatment timing | Start after surgery, POD | 54 [43-66]; range 25-93 |
| Treatment duration | Duration, days | 153 [112-154]; range 42-160 |
| Regimen | S-1 | 90 (39.3%) |
| Regimen | Gemcitabine | 53 (23.1%) |
| Regimen | Gemcitabine plus capecitabine | 47 (20.5%) |
| Regimen | mFOLFIRINOX | 36 (15.7%) |
| Regimen | 5-FU/leucovorin | 3 (1.3%) |
| Completion | Completed planned course | 152 (66.4%) |
| Completion | Stopped for documented recurrence | 20 (8.7%) |
| Completion | Early discontinuation because of toxicity | 21 (9.2%) |
| Completion | Early discontinuation because of frailty/recovery | 12 (5.2%) |
| Completion | Patient preference | 11 (4.8%) |
| Completion | Logistical or financial barrier | 7 (3.1%) |
| Completion | Regimen switch without planned-course completion | 6 (2.6%) |

Continuous values are median [IQR] unless otherwise specified. Regimen and completion percentages use the 229 treated patients as the denominator.

**Supplementary Table S8. Timing of postoperative biomarker assessments relative to adjuvant chemotherapy.**

| **Assessment** | **Postoperative day** | **Treatment phase at assessment** |
| --- | --- | --- |
| 1-month assessment | 31 [29-34]; range 22-44 | Pre-adjuvant: 216 (80.6%); on adjuvant: 13 (4.9%); no adjuvant: 39 (14.6%) |
| 3-month assessment | 89 [83-94]; range 75-105 | On adjuvant: 212 (79.1%); pre-adjuvant: 16 (6.0%); post-adjuvant: 1 (0.4%); no adjuvant: 39 (14.6%) |

**Supplementary Table S9. Bootstrap internal validation of the early-recurrence models.**

| **Model** | **N** | **Apparent AUC** | **Corrected AUC** | **Apparent Brier** | **Corrected Brier** | **Bootstrap intercept** | **Bootstrap slope** | **Successful resamples** |
| --- | --- | --- | --- | --- | --- | --- | --- | --- |
| Clinical | 255 | 0.648 | 0.605 | 0.192 | 0.204 | -0.212 | 0.749 | 1000 |
| Clinical + dynamic pattern | 255 | 0.702 | 0.661 | 0.181 | 0.194 | -0.168 | 0.788 | 1000 |

**Supplementary Table S10. Fine-Gray competing-risk sensitivity analysis for documented recurrence.**

| **Characteristic** | **sHR (95% CI)** | **P value** |
| --- | --- | --- |
| Age, per 10-year increase | 1.07 (0.89-1.27) | 0.476 |
| Tumor size, per 1-cm increase | 0.88 (0.71-1.08) | 0.221 |
| Pathologic node-positive disease | 1.27 (0.88-1.84) | 0.197 |
| Lymphovascular invasion | 0.98 (0.68-1.41) | 0.899 |
| R1 margin | 1.53 (0.99-2.37) | 0.055 |
| Preoperative CA19-9, per log2 increase | 1.11 (1.00-1.22) | 0.044 |
| Dynamic pattern: intermediate vs favorable | 1.37 (0.93-2.01) | 0.114 |
| Dynamic pattern: poor vs favorable | 2.15 (1.28-3.60) | 0.004 |

Recurrence was the event of interest and death before documented recurrence was the competing event (140 recurrence events and 6 competing deaths among 255 patients).

**Supplementary Table S11. Sensitivity analyses additionally adjusted for adjuvant chemotherapy status by POD 105.**

| **Outcome** | **Characteristic** | **Adjusted effect estimate (95% CI)** | **P value** |
| --- | --- | --- | --- |
| Early recurrence | Age, per 10-year increase | 1.24 (0.87-1.76) | 0.238 |
| Early recurrence | Tumor size, per 1-cm increase | 0.84 (0.59-1.19) | 0.319 |
| Early recurrence | Pathologic node-positive disease | 1.36 (0.73-2.53) | 0.331 |
| Early recurrence | Lymphovascular invasion | 1.22 (0.66-2.26) | 0.535 |
| Early recurrence | R1 margin | 1.72 (0.79-3.73) | 0.171 |
| Early recurrence | Preoperative CA19-9, per log2 increase | 1.32 (1.12-1.56) | 0.001 |
| Early recurrence | Dynamic pattern: intermediate vs favorable | 1.99 (1.00-3.99) | 0.052 |
| Early recurrence | Dynamic pattern: poor vs favorable | 4.30 (1.79-10.33) | 0.001 |
| Early recurrence | Adjuvant chemotherapy by POD 105 | 0.44 (0.20-0.94) | 0.034 |
| RFS | Age, per 10-year increase | 1.08 (0.90-1.30) | 0.390 |
| RFS | Tumor size, per 1-cm increase | 0.86 (0.70-1.05) | 0.129 |
| RFS | Pathologic node-positive disease | 1.27 (0.89-1.81) | 0.187 |
| RFS | Lymphovascular invasion | 1.05 (0.74-1.49) | 0.792 |
| RFS | R1 margin | 1.47 (0.94-2.29) | 0.088 |
| RFS | Preoperative CA19-9, per log2 increase | 1.12 (1.02-1.23) | 0.014 |
| RFS | Dynamic pattern: intermediate vs favorable | 1.34 (0.91-1.96) | 0.134 |
| RFS | Dynamic pattern: poor vs favorable | 1.73 (1.05-2.86) | 0.032 |
| RFS | Adjuvant chemotherapy by POD 105 | 0.48 (0.32-0.73) | <0.001 |
| OS | Age, per 10-year increase | 1.12 (0.91-1.38) | 0.269 |
| OS | Tumor size, per 1-cm increase | 0.90 (0.73-1.11) | 0.323 |
| OS | Pathologic node-positive disease | 1.28 (0.86-1.91) | 0.215 |
| OS | Lymphovascular invasion | 1.09 (0.74-1.62) | 0.660 |
| OS | R1 margin | 1.69 (1.04-2.73) | 0.034 |
| OS | Preoperative CA19-9, per log2 increase | 1.15 (1.04-1.27) | 0.008 |
| OS | Dynamic pattern: intermediate vs favorable | 1.52 (0.98-2.34) | 0.062 |
| OS | Dynamic pattern: poor vs favorable | 2.08 (1.21-3.57) | 0.008 |
| OS | Adjuvant chemotherapy by POD 105 | 0.51 (0.33-0.81) | 0.004 |

The early-recurrence model reports odds ratios; RFS and OS models report hazard ratios. All documented adjuvant chemotherapy starts among treated patients occurred before POD 105.

# Supplementary Figures

**Supplementary Figure S1. Missing laboratory measurements in the full eligible cohort. Bars show genuine missing measurements and labels show n (%); structurally non-applicable fields were not counted as missing data.**

**Supplementary Figure S2. Natural cubic spline analysis of the 3-month/1-month CA19-9 ratio and adjusted odds of early recurrence in the primary dynamic-pattern cohort. The solid line represents the adjusted odds ratio and the shaded band the 95% confidence interval.**

**Supplementary Figure S3. Alluvial plot linking postoperative CA19-9 state, 3-month inflammatory-nutritional recovery, the composite dynamic pattern, and 12-month early recurrence in the primary dynamic-pattern cohort.**
